# Expression of Endothelial Nitric Oxide Synthase and Endothelin-1 in Skin Tissue from Amputated Limbs of Patients with Complex Regional Pain Syndrome

**DOI:** 10.1155/2008/680981

**Published:** 2008-07-20

**Authors:** J. George Groeneweg, Claudia Heijmans Antonissen, Frank J. P. M. Huygen, Freek J. Zijlstra

**Affiliations:** ^1^Pain Treatment Center, Department of Anesthesiology, Erasmus Medical Center, P.O. Box 2040, 3000 CA Rotterdam, The Netherlands; ^2^Experimental Anesthesiology, Department of Anesthesiology, Erasmus Medical Center, P.O. Box 2040, 3000 CA Rotterdam, The Netherlands

## Abstract

*Background and Objectives*. Impaired microcirculation during the chronic stage of complex regional pain syndrome (CRPS) is related to increased vasoconstriction, tissue hypoxia, and metabolic tissue acidosis in the affected limb. Endothelial dysfunction is suggested to be the main cause of diminished blood flow. The aim of this study was to examine the distribution of endothelial nitric oxide synthase (eNOS) and endothelin-1(ET-1) relative to vascular density represented by the endothelial marker CD31-immunoreactivity in the skin tissue of patients with chronic CRPS. *Methods*. We performed immunohistochemical staining on sections of skin specimens obtained from the amputated limbs (one arm and one leg) of two patients with CRPS. *Results*. In comparison to proximal specimens we found an increased number of migrated endothelial cells as well as an increase of eNOS activity in distal dermis specimens. *Conclusions*. We found indications that endothelial dysfunction plays a role in chronic CRPS.

## 1. INTRODUCTION

Complex regional pain syndrome 1 (CRPS) is defined as “a syndrome in which the central nervous system representations of the somatosensory, somatomotor, and sympathetic systems are altered concomitantly with important peripheral changes such as edema, signs of inflammation, sympathetic-afferent coupling, and trophic changes” [1]. The manner in which the peripheral and central changes interact is only partly understood [1]. During the chronic stage of CRPS, increased vasoconstriction [2], tissue hypoxia [3], and metabolic tissue acidosis [4, 5] indicate that microcirculation is impaired, which affects the nutritive blood flow in superficial and deep tissues [6, 7].

The endothelium modulates vascular tone by releasing endothelium-derived vasodilators including nitric oxide (NO), prostacyclin, bradykinin, and endothelium-derived hyperpolarizing factor; and vasoconstrictors, such as endothelin-1 (ET-1) and angiotensin II, in response to a number of biochemical and physical stimuli [8]. Growing evidence [9] suggests that endothelial dysfunction is the main cause of diminished blood flow in chronic cold CRPS.

Although numerous papers about CRPS have been published since its first description in 1545 [10], literature regarding the associated pathological alterations in blood vessels is scarce. The few papers on this topic describe clearly visible abnormalities of the entire microvascular system, including an increase in the number of capillaries [11, 12], endothelial swelling, and changes in the vessel laminal wall [13]. The impressive capillary changes range from severely thickened basal membrane with intimal vacuolization, perivascular edema, and debris from pericytes between the basal membrane layers, to necrosis [14, 15]. Greatly thickened multilaminated walls were found, considerably reducing the inner diameter of the vessel [12, 16]. Endothelial cells with a shrunken appearance and capillaries with only endothelial cell debris in the lumina were observed, while other capillaries could be traced by the thickened basal membrane only, lacking the presence of other cellular remnants [15].

Histochemical procedures [17] and immunofluorescence [16] have been used to investigate the distribution of cutaneous nerve fibers, but there are no reports of using immunohistochemical staining to evaluate endothelial dysfunction in CRPS. Key elements in the function of the endothelium are NO and ET-1. The loss of endothelium-dependent NO mediated vasodilation occurs early during endothelial dysfunction [18]. Since NO has a half-life time of only 3–5 seconds [19], we measured endothelial nitric oxide synthase (eNOS), which is a constitutional endothelial cell enzyme that produces NO from L-arginine. Increased production and/or activity of ET-1 may participate in several pathologic states related to a dysfunctional endothelium [20].

The aim of this study was to examine the distribution of eNOS and ET-1 in relation to vascular density represented by the endothelial marker CD31-immunoreactivity in skin tissue from amputated limbs of patients with CRPS.

## 2. METHODS

### 2.1. Patients

Human tissue was obtained from the surgically amputated extremities of two patients diagnosed with CRPS type 1, according to the Bruehl diagnostic criteria [21], at the Pain Treatment Centre of the Erasmus MC. Both patients gave written informed consent prior to the amputation. A lower limb was amputated in patient A and an upper limb in patient B. Skin samples, harvested immediately following the amputations, were taken from the dorsal side of the foot of patient A and from the dorsal side of the hand of patient B; both of these samples were categorized as distal tissue samples. Proximal tissue samples were harvested from near the cutting face, taking tissue that appeared to be the least diseased.

Patient A was a 46-year-old female who developed CRPS in her right leg after an electromyography recording in 1996. One year later, symptoms appeared in the left leg, and in 1998, in her right hand. The patients’ complaints persisted and despite intense rehabilitation, both legs and the right hand became nonfunctional. There was edema in both legs, and there were severe contractures and pain in the legs and hand. Walking was impossible and she relied on a wheelchair for transport. During a visit to our clinic in 2003, muscle force, reflexes, and coordination could not be tested and there was hyperesthesia in both legs and the hand. She was treated with paracetamol, amitriptyline, durogesic, mannitol, and baclofen, and received an epidural block with marcaine and fentanyl. Due to continuous infections (erisipelas) in both legs in June 2003, antibiotics were prescribed but the infections were resistant to therapy. Therefore, the left leg had to be amputated in October 2003, followed by the right leg one week later. Postoperative healing was complicated by pressure ulcers and she was released from the hospital using paracetamol and tramadol for pain control. A video thermographic recording was not made, but the examining physician described both legs as very cold.

Patient B was a 38-year-old female who developed CRPS in the right hand in December 2002 as a consequence of a metacarpal II fracture. After 10 days in a plaster cast, she showed severe symptoms of CRPS with pain, edema, and contractures. Despite extensive pharmacotherapy to reduce pain and inflammation, improve peripheral blood flow, and relieve the spasticity, the patient developed severe dystonia in the right hand. The dystonia and pain made conventional nail care impossible; therefore, nail clipping was performed under general anesthesia every few months. The patient had contractures in her wrist and fingers, spasms of the musculature, burning pain, allodynia and hyperalgesia, edema, increased transpiration, excessive hair growth, and a darker skin color on the affected limb. A video thermographic recording [22, 23] showed that the CRPS extremity was 6.5°C colder than the contralateral unaffected control. Shoulder functioning was normal. Spinal cord trial stimulation in February 2004 was not successful. At the request of the patient, who could no longer cope with the burden of frequent general anesthesia, upper arm amputation was performed in May 2006. There were no postoperative complications and the patient experienced a dramatic reduction in pain.

### 2.2. Immunohistochemical staining for CD31 and eNOS

In order to demonstrate the vascular state of the CRPS skin tissues, the endothelial marker CD31 was visualized by immunoreactive (IR) staining.

Frozen skin tissue samples were cut in serial 6 *μ*m sections, transferred to poly-L-lysine-coated microscope slides (Menzel-Glaser, Omnilabo, Breda, the Netherlands), air dried, and stored at −80°C. For immunohistochemical staining, sections were thawed, fixed in acetone for 10 minutes, and rinsed with phosphate buffer saline (PBS, pH 7.8). The staining procedure was conducted in a half-automatic stainer (Sequenza, Shandon Scientific, Zeist, the Netherlands). The slides were incubated with 10% normal goat serum (NGS; Sanquin, Amsterdam, the Netherlands) for 10 minutes and then with primary mouse antihuman antibodies against CD31 (JC/70A Dako, Glostrup, Denmark) or eNOS (SA-258 BIOMOL International L.P., Exeter, UK) for 60 minutes. Both antibodies had been diluted in 1% blocking buffer (Blocking Reagent, Roche Diagnostics GmbH, and Mannheim, Germany). After each incubation step, the slides were rinsed with PBS for 5 minutes. After incubation with the primary antibodies, sections were rinsed and incubated with biotinylated goat antimouse antibodies (BioGenex, Klinipath, Duiven, the Netherlands) and 10% normal human serum (NHS; Sanquin) for 30 minutes. This step was followed by incubation with alkaline phosphatase-conjugated streptavidin (Biogenex, Klinipath) and 10% NHS for another 30 minutes. Slides were rinsed with both PBS and TRIS buffers (TRIS HCl 0.1 mol/L, pH 8.5), and then incubated with new fuchsine substrate (Chroma, Kongen, Germany) diluted in TRIS buffer. Finally, the sections were washed with PBS, counterstained with Gill’s hematoxylin (Merck, Darmstadt, Germany) for 30 seconds, rinsed with tap water, dried, and embedded in VectaMount (Vector, Burlingame, Calif, USA).

### 2.3. Immunohistochemical double staining for ET-1 and CD31

After fixing in acetone and washing with PBS, endogenous peroxidase was blocked with 0.1% sodium azide and 0.03% hydrogen peroxide in PBS for 30 minutes. Sections were rinsed and incubated with 10% NGS for 10 minutes, followed by incubation with rat antihuman ET-1 (3G10, R&D systems, Abingdon, UK) for 60 minutes. The sections were rinsed and incubated with goat antirat antibody conjugated with alkaline phosphatase (Sigma, St. Louis, Mo, USA) and 10% NHS for 30 minutes. Thereafter, the slides were rinsed and incubated with mouse antihuman CD31 for 60 minutes. Then sections were rinsed and incubated with biotinylated goat antimouse antibodies and 10% normal human serum (NHS) and 10% normal rat serum (Sanquin) for 30 minutes. After washing, the slides were incubated with streptavidin-conjugated peroxidase (Biogenex, Klinipath) and 10% NHS for another 30 minutes. After rinsing with PBS and substrate TRIS buffer, slides were incubated for 30 minutes in fast blue substrate (Sigma). Finally, sections were rinsed and incubated with peroxidase nova red substrate (Vector) for 5 minutes, rinsed with PBS, and embedded in VectaMount.

### 2.4. Quantification of the immunoreaction

ENOS and CD31 staining was performed on adjacent slides of serial sections. Slides were examined using a Leica microscope fixed with a Leica DC500 camera for digitizing images. Semiquantitative evaluation of the different markers was performed by counting the number of positive blood vessels in the dermis of two sections each from both the distal and proximal specimens. After measuring the total area of the dermis using the Leica imaging analysis system, the number of positive blood vessels per square millimeter was calculated. In the case of ET-1 and CD31 double staining, we determined the number of ET-1 positive cells in the dermis and the percentage of positive ET-1 blood vessels on the total number of blood vessels.

## 3. RESULTS

### 3.1. Vascular status in CRPS

**Figure 1:**
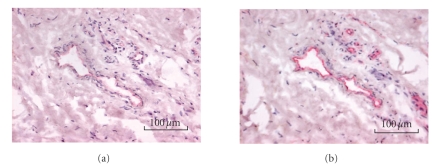
(a) CD31-immunoreactive vessels in skin tissue from the amputated arm of CRPS patient A. CD31-positive blood vessels are stained red. (b) eNOS staining of a serial section. eNOS-positive blood vessels are stained red.

The qualitative differences between the distal and proximal specimens are shown in Figure 1(a). These differences were confirmed by qualitative image analysis of the sections. In both patients, endothelial immunoreactivity was more prominent in distal than in proximal specimens. In patient A, the mean number of CD31-IR capillaries in distal tissue was 43 blood vessels/mm^2^ versus 14 blood vessels/mm^2^ in proximal tissue; in patient B, the means were 39 and 19 blood vessels/mm^2^, respectively.

### 3.2. eNOS immunoreactivity

eNOS immunoreactivity of capillaries and other small-diameter blood vessels was prominent in the distal specimens (see Figure 1(b)). The measure of eNOS-IR endothelium in patient A was 23 blood vessels/mm^2^ in distal tissue versus 7 blood vessels/mm^2^ in proximal tissue; in patient B, the means were 20 and 13 blood vessels/mm^2^, respectively.

### 3.3. Ratios

The ratios of eNOS/CD31-IR vessels were similar. In patient A, the mean ratios were 53% and 50% in distal and proximal tissues, respectively, whereas in patient B, the mean ratios were 51% and 68%, respectively.

### 3.4. Endothelin-1 positive cells

**Figure 2:**
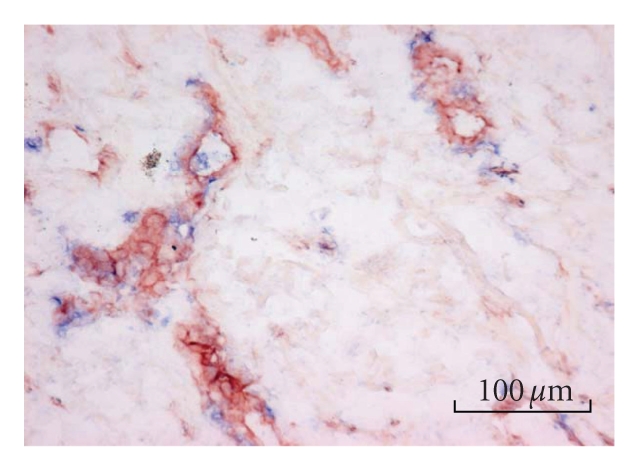
Double staining for CD31 and ET-1 in skin tissue from the amputated leg of CRPS patient B. CD31-positive blood vessels are stained red and ET-1-positive cells are stained blue.

The mean number of ET-1-positive cells was determined in both the dermis and the blood vessels (Figure 2). In patient A, there were 81 ET-1-positive dermis cells/mm^2^ in distal tissue versus 22 ET-1-positive dermis cells/mm^2^ in proximal tissue; in patient B, there were 42 and 12 ET-1-positive dermis cells/mm^2^, respectively. The mean percentages ET-1-positive blood vessels were similar; in patient A, there were 70% ET-1-positive blood vessels in the distal tissue and 69% in the proximal tissue; in patient B, the mean values were 78% and 63%, respectively.

## 4. DISCUSSION

Because of the increased risk of an overreaction to a skin biopsy [15], we could not take skin tissue samples from CRPS-affected limbs in the usual manner. Therefore, we used specimens taken from amputated limbs, which are seldom available. Proximal and distal specimens from the amputated leg or arm from two different patients were dissected out immediately after the amputation, and deep frozen in liquid nitrogen. As specimens from the contra-lateral side or healthy tissue were not available for comparison, we considered the distal specimens as the most-affected and the proximal specimens as non- or least-affected tissue. This is the first study that investigates the distribution of eNOS and ET-1 in tissue of CRPS patients, therefore we decided to limit the study to skin tissue, following previous observations in skin blister fluid obtained from CRPS patients. Expanding the study to muscle and/or nerve tissue might have provided additional insights in mechanisms underlying CRPS.

Tissue blood distribution is altered in patients with CRPS. It is generally accepted that during the course of this disease, and partly due to disuse, tissue ischemia will occur, leading to chronic pain [24]. However, until now, there was no evidence suggesting that these patients have endothelial dysfunction or impaired angiogenesis in response to ischemia. We observed regional differences in the expression of endothelial markers in the CRPS-affected limbs. Information about the normal distribution of these markers between proximal and distal upper or lower extremities is not available. Expression of CD31, eNOS, and ET-1 was highest in the distal specimens, representing the most affected samples of the diseased limbs. However, the eNOS/CD31 ratios were similar, ranging from 51% to 68%. Palatka et al. [25] reported an eNOS/CD31 ratio of 92% in healthy mucosal biopsies, whereas this ratio was 8% in tissue samples from patients with Crohn’s disease and 82% in samples from patients with ulcerative colitis, which suggests that eNOS activity is diminished in disease-affected endothelial cells. Although values from mucosa may not be comparable to the skin tissue values in our study, the suggestion is made that eNOS activity was diminished in our patients, but not dramatically. Moreover, we observed no distinct regional differences, coinciding with the observations that during the course of the disease, pain spreads through the entire limb and that CRPS may also occur in the contra lateral limb and/or the other extremities [26]. It has been shown that during hypoxia, expression of both endothelial cell markers CD31 and eNOS increases [27]. In the case of ischemia, eNOS is essential for promoting collateral growth in order to restore blood distribution to the tissues [28, 29]. Therefore, in patients with CRPS, supplementing NO precursors will not provide benefit because eNOS activity is compromised. Treating patients with chronic CRPS with an endothelium-independent NO donor might be a more effective strategy [30].

Our data confirmed the previously reported increase in capillary density in CRPS-affected tissue [11, 12], and showed a higher number of ET-1-positive blood vessels/mm^2^ in distal versus proximal specimens. There were marked differences (3 to 4 fold) in the number of ET-1-positive cells between the distal and proximal dermis. ET-1 promotes proliferation and migration of endothelial cells via endothelin-B receptors [31]. These migrated cells are associated with swelling and laminal wall disruptions [13]. The pronounced increase in ET-1 expression in the distal limbs confirms earlier work by our group in which we found elevated levels of ET-1 in the superficial blister fluids harvested from the distal region of CRPS limbs [24]. However, alterations in the number of ET-1-positive cells and skin blister fluid concentrations do not necessarily reflect an increase in the number of endothelin-B receptor-binding CD31-positive endothelial cells [31]. Nevertheless, ET-1 blockers could provide relief.

This study was limited to only two CRPS type 1 patients, and lacks appropriate control in the form of contralateral tissue, or a normal distribution of these markers. Both patients were chronic and have been treated with many medical and interventional procedures. Conclusions can therefore only apply to potential mechanisms that maintain chronic CRPS, like severe vasoconstriction, blood supply redistribution due to abnormal blood flow shunting with hypoperfusion in nutritive vessels, hypoxia, lactate increase, and acidosis [1].

In conclusion, we found an indication that endothelial dysfunction plays a role in chronic CRPS. In comparison to proximal specimens, we found an increased number of migrated endothelial cells as well as an increase of eNOS activity in distal dermis specimens.
